# PW01-019 – MEFV gene mutations in 53 periodic fever patients

**DOI:** 10.1186/1546-0096-11-S1-A72

**Published:** 2013-11-08

**Authors:** N Toplak, M Debeljak, T Avcin

**Affiliations:** 1 University Children's Hospital Ljubljana, Department of Allergology, Rheumatology and Clinical Immunology, University Medical Centre Ljubljana, Slovenia; 2 Genetic Laboratory, University Children's Hospital Ljubljana, University Medical Centre Ljubljana, Slovenia; 3 Department of Allergology, Rheumatology and Clinical Immunology, University Children's Hospital Ljubljana, University Medical Centre Ljubljana, Ljubljana, Slovenia

## Introduction

FMF is a very rare disease in Slovenia and so far only few cases were diagnosed. It is known that environment influences the clinical picture of FMF.

## Objectives

We were interested if patients with PFAPA or PFAPA like clinical picture and patients with other periodic fevers followed in single tertiary centre at University Children’s hospital in Ljubljana, Slovenia, have mutations in *MEFV* gene.

## Methods

We collected clinical and laboratory data from periodic fever patients followed at our center from the beginning of 2006 to the end of 2012. Results of genetic testing for *MEFV* gene mutations were also collected. Genetic testing was performed in Genetic laboratory of University Children’s Hospital Ljubljana. All 10 exons and exon/intron regions of *MEFV* gene were directly sequenced with ABI Prism 310 Genetic analyzer.

## Results

From 2006 86 patients with periodic fevers were followed at our center; 4 adults and 82 children. The majority of them (80%) were diagnosed with PFAPA syndrome according to Marshall criteria. Genetic testing for *MEFV* mutations was performed in 53 patients (60%). In 20 (38%) patients no mutations were found (N/N). Twenty one patient was found to have R202Q/N genotype (two had clinical presentation of FMF) and another 6 were found to be homozygous for R202Q mutation (all with PFAPA phenotype). E148Q/R202Q was found in one patient with PFAPA phenotype. P369S/R408Q and R202Q were found in three patients. Among them one adult had undefined periodic fever and two children were having PFAPA phenotype with abdominal pains during attacks. All together 31 patients (58%) were found to have at least one R202Q mutation present in exon 2 of *MEFV* gene. One M694V/N and one K695R/N genotype was found in two patients with FMF phenotype.

One patient with R202Q/N genotype and negative genetic testing of exon 3 in *NLRP* gene was having clinical presentation of CAPS like syndrome and in one adult with TRAPS clinical picture R92Q mutation in *TNFRSF1A* gene was found and no mutations in *MEFV* gene.

To test if R202Q mutation is more common in periodic fever patients than in apparently healthy population we also tested 50 apparently healthy persons for R202Q. Twenty nine (64%) were positive for at least one R202Q mutation.

## Conclusion

In our cohort of periodic fever patients, 33 patients (62%) were found to have mutations in MEFV gene. Majority of them were positive for R202Q mutation, mostly with PFAPA phenotype. The percentage of positivity for R202Q mutation was not higher than in apparently healthy population. We haven’t found a single patient with typical clinical picture of FMF and 2 mutations. Two patients with typical clinical picture were heterozygous for *MEFV* gene mutations and two for R202Q mutation which is, according to our results, a polymorphism.

## Disclosure of interest

None declared

